# Light Suppresses Bacterial Population through the Accumulation of Hydrogen Peroxide in Tobacco Leaves Infected with *Pseudomonas syringae* pv. *tabaci*

**DOI:** 10.3389/fpls.2016.00512

**Published:** 2016-04-21

**Authors:** Dan-Dan Cheng, Mei-Jun Liu, Xing-Bin Sun, Min Zhao, Wah S. Chow, Guang-Yu Sun, Zi-Shan Zhang, Yan-Bo Hu

**Affiliations:** ^1^College of Life Science, Northeast Forestry UniversityHarbin, China; ^2^State Key Laboratory of Crop Biology, College of Life Sciences, Shandong Agricultural UniversityTai’an, China; ^3^Division of Plant Science, Research School of Biology, The Australian National University, CanberraACT, Australia

**Keywords:** light, *Nicotiana tabacum*, photosystems, *Pseudomonas syringae* pv. *tabaci*, reactive oxygen species

## Abstract

*Pseudomonas syringae* pv. *tabaci* (*Pst*) is a hemibiotrophic bacterial pathogen responsible for tobacco wildfire disease. Although considerable research has been conducted on the tobacco plant’s tolerance to *Pst*, the role of light in the responses of the photosystems to *Pst* infection is poorly understood. This study aimed to elucidate the underlying mechanisms of the reduced photosystem damage in tobacco leaves due to *Pst* infection under light conditions. Compared to dark conditions, *Pst* infection under light conditions resulted in less chlorophyll degradation and a smaller decline in photosynthetic function. Although the maximal quantum yield of photosystem II (PSII) and the activity of the photosystem I (PSI) complex decreased as *Pst* infection progressed, damage to PSI and PSII after infection was reduced under light conditions compared to dark conditions. *Pst* was 17-fold more abundant in tobacco leaves under dark compared to light conditions at 3 days post inoculation (dpi). Additionally, H_2_O_2_ accumulated to a high level in tobacco leaves after *Pst* infection under light conditions; although to a lesser extent, H_2_O_2_ accumulation was also significant under dark conditions. Pretreatment with H_2_O_2_ alleviated chlorotic lesions and decreased *Pst* abundance in tobacco leaves at 3 dpi under dark conditions. MV pretreatment had the same effects under light conditions, whereas 3-(3,4-dichlorophenyl)-1,1-dimethylurea pretreatment aggravated chlorotic lesions and increased the *Pst* population. These results indicate that chlorotic symptoms and the size of the bacterial population are each negatively correlated with H_2_O_2_ accumulation. In other words, light appears to suppress the *Pst* population in tobacco leaves through the accumulation of H_2_O_2_ during infection.

## Introduction

Plants are simultaneously exposed to abiotic and biotic stresses, and their responses to pathogens (bacteria, fungi, and viruses) at various stages of development are largely dependent on environmental factors such as light, temperature, and water (Bostock et al., 2014; Suzuki et al., 2014). Light is the major external factor influencing plant growth, development and physiology, and it also has an important impact on defense responses as follows: (i) by providing signals for the deployment of defensive barriers; (ii) by influencing the general energy supply and, thus, the ‘fuel’ available to launch and sustain responses against invaders; and (iii) by inducing the production of ROS in chloroplasts and peroxisomes (Kangasjärvi et al., 2012). Light availability is particularly important during the first hours after infection, as the absence of light during the early plant–pathogen interaction stages negatively affects development of the hypersensitive response (HR) at later stages (Griebel and Zeier, 2008).

It is well established that light is required for regulating resistance responses in several plant–pathogen interactions (Roberts and Paul, 2006; Delprato et al., 2015). For example, Cerrudo et al. (2012) revealed that a low red/far-red light ratio reduces resistance to the necrotrophic fungus *Botrytis cinerea* in *Arabidopsis*. Mühlenbock et al. (2008) showed that plants acclimate to excessive excitation energy (a condition in which the amount of light absorbed by the photosystems exceeds that required for photosynthetic metabolism) and display increased resistance to a virulent strain of *Pseudomonas syringae*. In addition, Zeier et al. (2004) reported light-dependent resistance of *Arabidopsis* to *P. syringae* pv. *tomato* DC3000 *(Pto)* conferred through a different resistance–avirulence gene interaction; they also observed that pathogenesis-related protein expression was light dependent in *Arabidopsis* plants inoculated with *P. syringae* pv. *maculicola* (Zeier et al., 2004). Genoud et al. (2002) demonstrated that light plays important roles in SA-mediated pathways and that it is required for the HR, and *Arabidopsis* plants grown in the dark exhibit compromised local and systemic resistance responses to *Pto*.

*Pseudomonas syringae* pv. *tabaci* (*Pst*) is a hemibiotrophic bacterial pathogen responsible for tobacco wildfire disease (Ramegowda et al., 2013). Previous research has shown that a circadian rhythm has a large impact on plant immunity during plant–pathogen interaction (Hua, 2013). For example, the susceptibility of tomato plants to *Pto* is influenced by a diurnal rhythm, with the greatest susceptibility in the evening (Yang et al., 2015). Light regulation of defense responses is relevant not only during artificial darkening but also during natural light/dark cycles (Kangasjärvi et al., 2012). Considerable research has been conducted on the tobacco plant’s tolerance to *Pst* (Hann and Rathjen, 2007; Taguchi and Ichinose, 2011; Lee et al., 2013) as well as on the photosynthetic performance of plants infected by other *P. syringae* pathovars (Bonfig et al., 2006; Rodríguez-Moreno et al., 2008). For example, Berger et al. (2007) found decreased maximum PSII quantum yield (*F*_v_*/F*_m_) in *Pto*-infected *Arabidopsis* leaves, and Pérez-Bueno et al. (2015) found decreased photochemical efficiency of PSII in *Pto-*infected *Phaseolus vulgaris* leaves. The effects of light on plants under various abiotic stresses have also been well studied. For example, Crous et al. (2012) reported light-induced inhibition of leaf respiration in field-grown *Eucalyptus saligna* under elevated atmospheric CO_2_ and drought, and De Frenne et al. (2015) revealed that light accelerates plant responses to warming. However, the underlying mechanisms by which light affects the tobacco–*Pst* interaction, especially when explored from a physiological perspective, remain poorly understood.

Reactive oxygen species are unavoidable by-products of photosynthesis in plants, and photosynthetic electron transfer reactions in light-exposed green tissues are a significant source of ROS due to the formation of highly reactive singlet oxygen (^1^O_2_) and the less reactive superoxide (O_2_^-^) and H_2_O_2_ which can potentially yield highly reactive hydroxyl free radicals (Aro et al., 2005; Li et al., 2009). The overall level of ROS in cells is the result of both ROS production and ROS scavenging. The main scavenging systems in plant cells include antioxidative enzymes and antioxidants (Mittler et al., 2011); among the former, CAT is thought to play a very important role (Zavaleta-Mancera et al., 2007).

A marked decline in photosynthesis will lead to excessive excitation energy which, if not dissipated safely, will increase the production of ROS in plant leaves (Jia et al., 2010). Despite their potential toxicity, ROS actually have a dual role *in vivo*, depending on their concentration, site and duration of action, as well as the plant’s previous exposure to stress (Miller et al., 2010). At low concentrations, ROS act as messenger molecules involved in signaling related to acclimation and trigger defense mechanisms against stress; at high concentrations, ROS promote programmed cell death, membrane lipid peroxidation and oxidative damage to the photosynthetic apparatus (Foyer and Noctor, 2009; Kangasjärvi et al., 2009; Galvez-Valdivieso and Mullineaux, 2010; Suzuki et al., 2012).

In this study, we aimed to investigate if the photosynthetic apparatus of tobacco leaves responds to *Pst* infection differently under light and dark conditions. What is the role of light during tobacco responses to *Pst* infection, and are ROS involved in this progress? To address these questions, we (1) evaluated changes in the activities of PSI and PSII, (2) monitored the content of H_2_O_2_ and the bacterial population, and (3) evaluated the relationship between light, H_2_O_2_ and the *Pst* population after *Pst* inoculation of tobacco leaves.

## Materials and Methods

### Plant Material and *Pst* Infiltration

Seeds of *Nicotiana tabacum* cv. Longjiang 911, a susceptible cultivar (Sun et al., 2011), were germinated in vermiculite; after 45 days, the seedlings were transplanted into pots containing a compost-soil substrate and grown in a greenhouse (25°C, 70% relative humidity) under long-day conditions (14 h light/10 h darkness). The two upper fully expanded attached leaves of 6- to 8- week-old plants were used for experiments.

*Pseudomonas syringae* pv. *tabaci* was grown overnight on solid King’s B agar plates (King et al., 1954) and diluted with distilled water to a concentration 10^5^ cfu ml^-1^. Distilled water (mock) or bacterial suspensions were hand-infiltrated using a needleless syringe into mesophyll tissue on the abaxial side of the leaves at 8 am. The infiltrated area was approximately 1 cm ×1 cm, and *in situ* measurements were performed at a distance of 0.5 cm from the infiltration point. Following inoculation, the leaves were kept under a 14 h light (200 μmol m^-2^ s^-1^)/10 h dark cycle or continuous darkness at 25°C in a chamber with 70% relative humidity.

To induce or inhibit the generation of H_2_O_2_ under light conditions, a solution of MV (1 μM) or DCMU (70 μM) was uniformly sprayed onto the tobacco leaves at 1 h prior to *Pst* infiltration. At the concentrations used, neither of the chemicals had a detectable effect on *Pst* growth. To increase the H_2_O_2_ content in tobacco leaves under dark conditions, exogenous H_2_O_2_ (4 mM) was uniformly sprayed onto tobacco leaves prior to *Pst* infiltration.

### Measurement of Spectral Reflectance in *Pst*-Infiltrated Zone of Tobacco Leaves

Spectral reflectance was measured by a Unispec SC field portable spectrometer (PP Systems, USA). Leaf reflectance was measured with a bifurcated fiber optic cable and a leaf clip (UNI410, PP Systems, USA). The leaf clip held the fiber optic cable at a 60° angle to the adaxial leaf surface (30° from the normal). Leaf illumination from a tungsten halogen lamp in the spectrometer was provided through one branch of the bifurcated fiber.

### Measurements of Total Chlorophyll Content in Tobacco Leaves after *Pst* Infection

Leaf disks (diameter 1 cm) within the region of infiltration were excised, and total chlorophyll was extracted with 80% acetone in the dark for 72 h at 4°C. The extracts were analyzed using a UV-visible spectrophotometer (UV-1601, Shimadzu, Japan) according to the method of Porra (2002).

### Measurements of the Chlorophyll *a* Fluorescence Transient (OJIP) and PSI Complex Activity in Tobacco Leaves after *Pst* Infection

The induction kinetics of prompt fluorescence and the MR_820nm_ were simultaneously recorded using a Multifunctional Plant Efficiency Analyzer, M-PEA (Hansatech, UK), as described (Strasser et al., 2010). At 3 dpi under light or dark conditions, all leaves were dark-adapted for 20 min before the measurements. Chlorophyll *a* fluorescence transients were analyzed using the JIP-test, as follows: *F*_v_/*F*_m_ = 1 - (*F*_o_/*F*_m_), where *F*_o_ and *F*_m_ are the initial and maximum fluorescence values of the induction kinetics curves, respectively.

### Detection of H_2_O_2_ Generation in Tobacco Leaves after *Pst* Infection

*In situ* H_2_O_2_ was detected by DAB staining, as described previously (Ueno et al., 2003). Leaf disks (diameter 1 cm) within the region of infiltration were excised and incubated in 1 mg ml^-1^ DAB-HCl (pH 5.5) for 6 h at room temperature; chlorophyll was removed with 95% ethanol. H_2_O_2_ was extracted and quantified according to the method of Patterson et al. (1984). Leaf segments (0.5 g) were ground in liquid nitrogen and extracted with 5 ml of 5% (w/v) trichloroacetic acid; the mixture was centrifuged at 16 000 × *g* for 10 min and the supernatant was collected. A titanium reagent (0.4 ml of 20 % titanic tetrachloride in concentrated HCl, v/v) was added to 3 ml of the extract followed by the addition of 0.6 ml of NH_4_OH to precipitate the peroxide-titanium complex. After 5 min of centrifugation at 12 000 × *g*, the supernatant was discarded, and the precipitate was solubilized in 2 ml of 2 M H_2_SO_4_. The absorbance of the final solution was measured at 415 nm. A standard curve was used to determine the concentration of H_2_O_2_ in the extract.

### Enzyme Assays

Leaf segments (0.5 g) of infiltrated areas at 3 dpi were ground to a fine powder with liquid nitrogen and then homogenized in 5 ml of 50 mM of potassium phosphate buffer (pH 7.8) containing 1 mM EDTA, 0.3 % Triton X-100 and 1% (w/v) polyvinylpyrrolidone. The homogenate was centrifuged at 13000 × *g* for 20 min at 4°C, and the supernatant was used for the following enzyme assays and protein measurement. SOD (EC 1.15.1.1) activity was assayed by monitoring inhibition of the photochemical reduction of nitro blue tetrazolium according to the method of Giannopolitis and Ries (1977). CAT (EC 1.11.1.6) activity was determined according to the method of Aebi (1984) by monitoring the decrease in absorbance at 240 nm as a result of decomposition of H_2_O_2_. The total soluble protein concentration was determined using the method of Bradford (1976) with bovine serum albumin as the standard.

### Enumeration of Bacteria in Tobacco Leaf Tissue after *Pst* Infection

To monitor bacterial propagation in the leaves, two leaf disks (diameter 1 cm) within the region of infiltration were excised from each plant at 8 am and homogenized in 1 ml of 10 mM MgCl_2_ (Katagiri et al., 2002). After appropriate dilution, the bacterial cfu was assessed by plating an appropriate dilution on solid King’s B agar plates followed by incubation at 25°C.

### *P. syringae* pv. *tabaci* Growth under Light or Dark Conditions

The direct effect of light on *Pst* growth was evaluated by plating the same amount of *Pst* sample on solid King’s B agar plates. The plates were then divided into two groups for cultivation for 3 days at 25°C under light (200 μmol m^-2^ s^-1^) or dark conditions. All bacteria from each group were collected, and the total number of *Pst* cells was counted to reflect the *Pst* multiplication rate.

### Chemicals Used in the Study

All the compounds used in this study were sourced from Sigma–Aldrich (USA).

### Statistical Analysis

Means (at least three independent measurements) were compared by analysis of variance (ANOVA) and LSD range test at a 5% level of significance.

## Results

### The Effect of Light on Spectral Reflectance in Tobacco Leaves after *Pst* Infection

**FIGURE 1:**
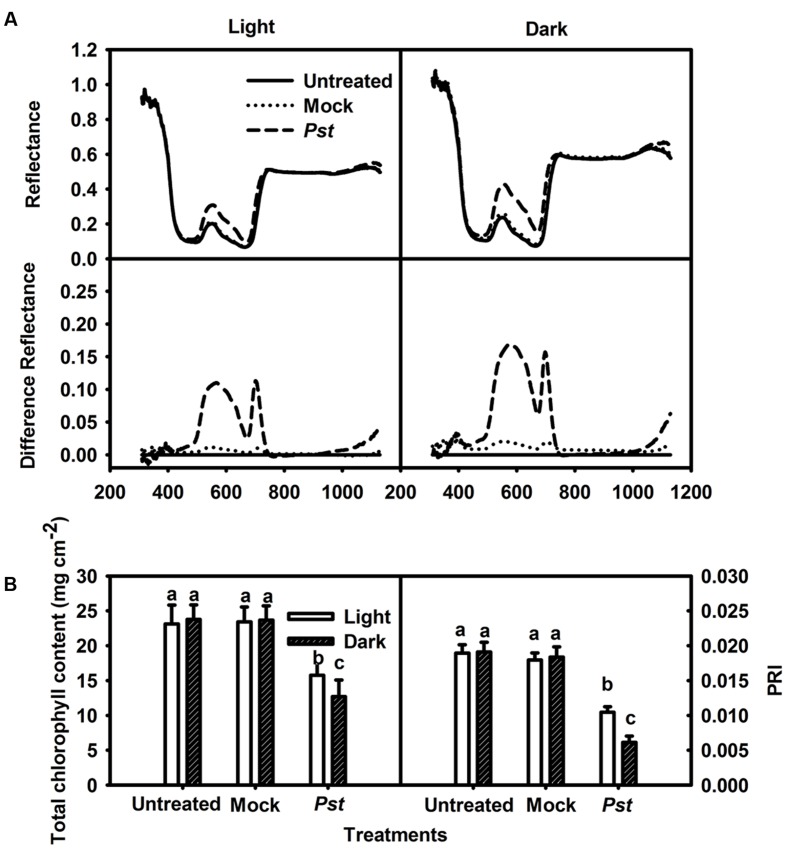
**Relative changes in spectral reflectance, total chlorophyll content and PRI after *Pst* infection of tobacco leaves.** **(A)** Relative changes in spectral reflectance in tobacco leaves at 3 dpi under light (200 μmol m^-2^ s^-1^) or dark conditions. The difference in reflectance was obtained by subtracting the kinetics of untreated leaves from the kinetics of tobacco leaves treated with distilled water (Mock - untreated) or *Pst* (*Pst*- untreated). Each curve represents the average of 50 independent measurements. **(B)** Relative changes in total chlorophyll content at 3 dpi in tobacco leaves. PRI was calculated from the spectral reflectance. Means ± SE of 50 replicates are shown. Different letters above the columns indicate significant differences between treatments at *P* < 0.05.

Spectral reflectance has been widely used to study the physiological status of plants under different environmental conditions. It has been shown that chlorophyll content influences the spectral reflectance in the visual waveband (500 - 700 nm) and that the inner structure of the leaf influences the spectral reflectance in the near-infrared waveband (700-1,300 nm) (Sims and Gamon, 2002). An established vegetation index, i.e., PRI, was calculated as PRI = (R531 - R570)/(R531 + R570). Although the spectral curves of the infiltrated zone revealed an increase in the visual waveband, there were insignificant changes in the near-infrared waveband at 3 dpi under light and dark conditions (**Figure 1A**). These results indicated that the chlorophyll content in the infiltrated zone of the tobacco leaves decreased markedly but that the inner structure was not severely damaged at 3 dpi under light and dark conditions. The total chlorophyll content in the infiltrated zone was then measured, showing decreases to 68.2 and 53.4% of the untreated values at 3 dpi under light and dark conditions, respectively (**Figure 1B**). PRI can serve as an estimate of the photosynthetic light use efficiency (Evain et al., 2004) and an indicator of photosynthetic function (Weng et al., 2009). PRI was found to have decreased to 55.2 and 32.1% at 3 dpi under light and dark conditions, respectively (**Figure 1B**). These results indicated that *Pst* infection in tobacco leaves led to more severe chlorophyll degradation and photosynthetic function decline under dark conditions than under light conditions.

### The Effect of Light on the PSI and PSII Activities in Tobacco Leaves after *Pst* Infection

**FIGURE 2:**
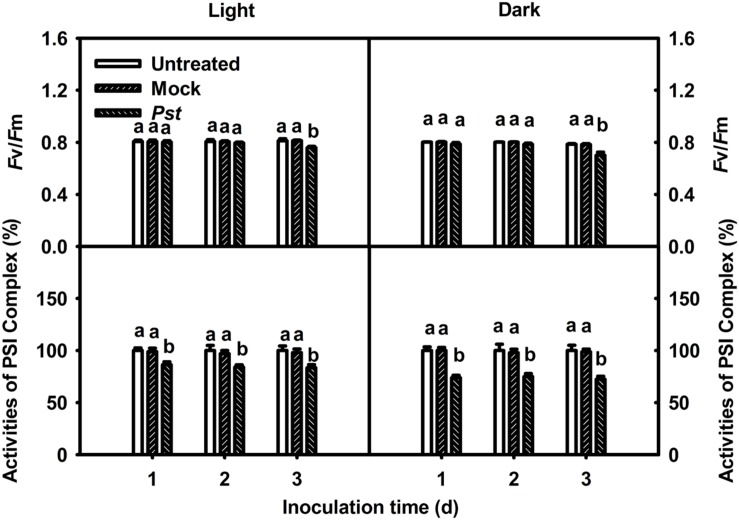
**Relative changes in *F*_v_/*F*_m_ and PSI complex activity after *Pst* infection of tobacco leaves.** Chlorophyll *a* fluorescence transients were analyzed with the JIP-test. The maximum quantum yield of PSII (*F*_v_/*F*_m_) and PSI complex activity values were calculated after tobacco leaves were inoculated with distilled water (Mock) or *Pst* for different durations under light (200 μmol m^-2^ s^-1^) or dark conditions. The initial PSI complex activity of untreated tobacco leaves was taken as 100%; the activities of mock- and *Pst*-treated leaves were calculated as the percentage of activity in untreated leaves. Means ± SE of 10 replicates are presented. Different letters above the columns indicate significant differences between treatments at *P* < 0.05.

The effect of *Pst* infection on the photosynthetic apparatus was evaluated at 3 dpi. The maximum quantum yield of PSII (*F*_v_/*F*_m_) decreased to 93.4 and 89.2% of the values of untreated leaves at 3 dpi under light and dark conditions, respectively (**Figure 2**). The MR_820nm_ signal provides information about the oxidation status of P700 in PSI, including the oxidation state of plastocyanin that donates electrons to PSI. The induction curve of MR_820nm_ for dark-treated leaves obtained by a saturating red light showed a fast oxidation phase and a subsequent reduction phase. The initial slope of the oxidation phase of MR_820nm_ indicates the capability of P700 to get oxidized, which is then used to reflect the activity of PSI (Oukarroum et al., 2013; Gao et al., 2014), and the PSI complex activities of the treated leaves were 83.5 and 72.4% of those of untreated leaves at 3 dpi under light and dark conditions, respectively (**Figure 2**). These results indicated that the extent of the decrease in *F*_v_/*F*_m_ and PSI complex activity in tobacco leaves was greater in darkness than under light conditions after *Pst* infection.

### The Effect of Light on the Bacterial Population in Tobacco Leaves at 3 dpi after *Pst* Infection

**FIGURE 3:**
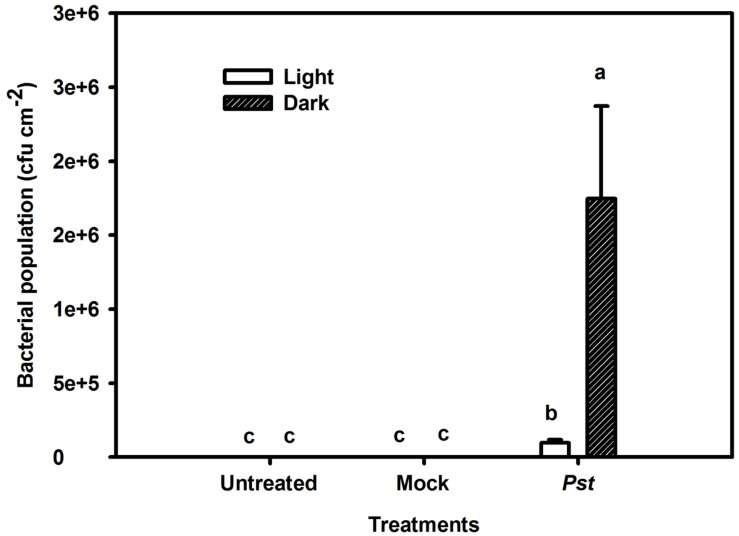
**Relative changes in the bacterial population after *Pst* infection in tobacco leaves.** Bacterial population was evaluated after leaves were inoculated with distilled water (Mock) or *Pst* at 3 dpi under light or dark conditions. Means ± SE of six replicates are presented. Different letters above the columns indicate significant differences between treatments at *P* < 0.05.

**FIGURE 4:**
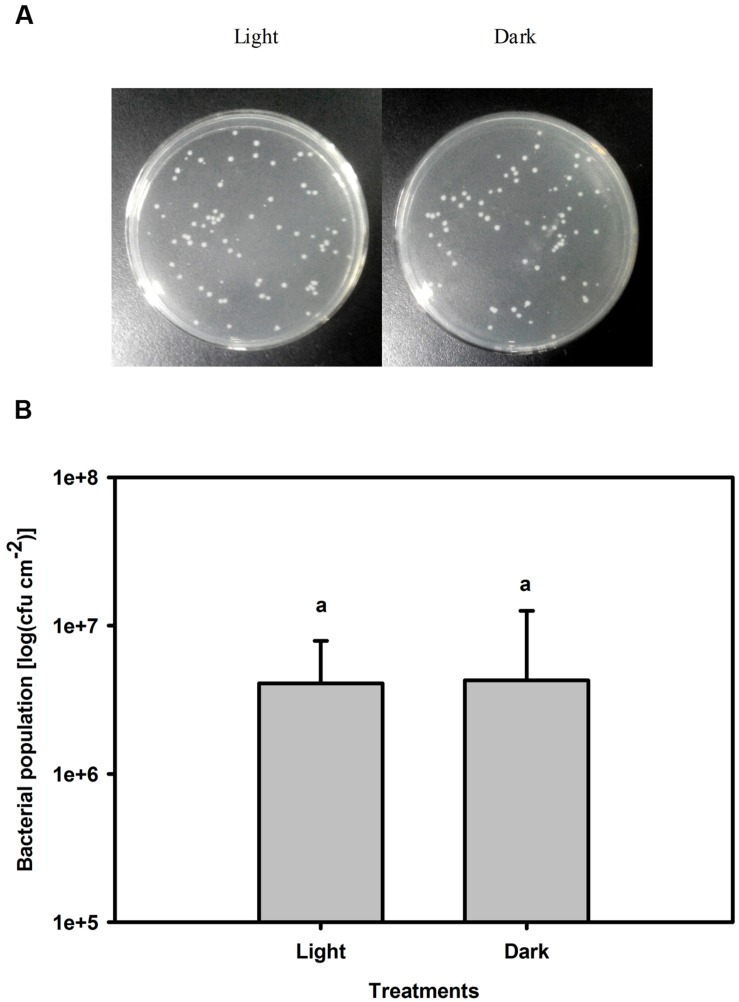
**Changes in the *Pst* population after 3 days of cultivation under light or dark conditions.** **(A)** *Pst* population was counted on solid King’s B agar plates after appropriate dilution. **(B)** *Pst* population was calculated after 3 days cultivation under light (200 μmol m^-2^ s^-1^) or dark conditions. Means ± SE of four replicates are presented. Different letters above the columns indicate significant differences between treatments at *P* < 0.05.

The population of *Pst* in leaves increased by several orders of magnitude at 3 dpi compared with that of untreated leaves. Furthermore, the bacterial population was 17-fold greater in darkness than under light conditions at 3 dpi (**Figure 3**). To exclude a direct effect of light on the multiplication rate of *Pst* during infection, this effect was evaluated on plate-grown bacteria (without leaves) at a constant temperature. No difference in the *Pst* population was observed between cultivation in light and dark conditions, indicating that light *per se* does not have a direct effect on *Pst* growth (**Figure 4**).

### Changes in H_2_O_2_ Levels and Antioxidative Enzyme Activities in Tobacco Leaves after *Pst* Infection under Light or Dark Conditions

**FIGURE 5:**
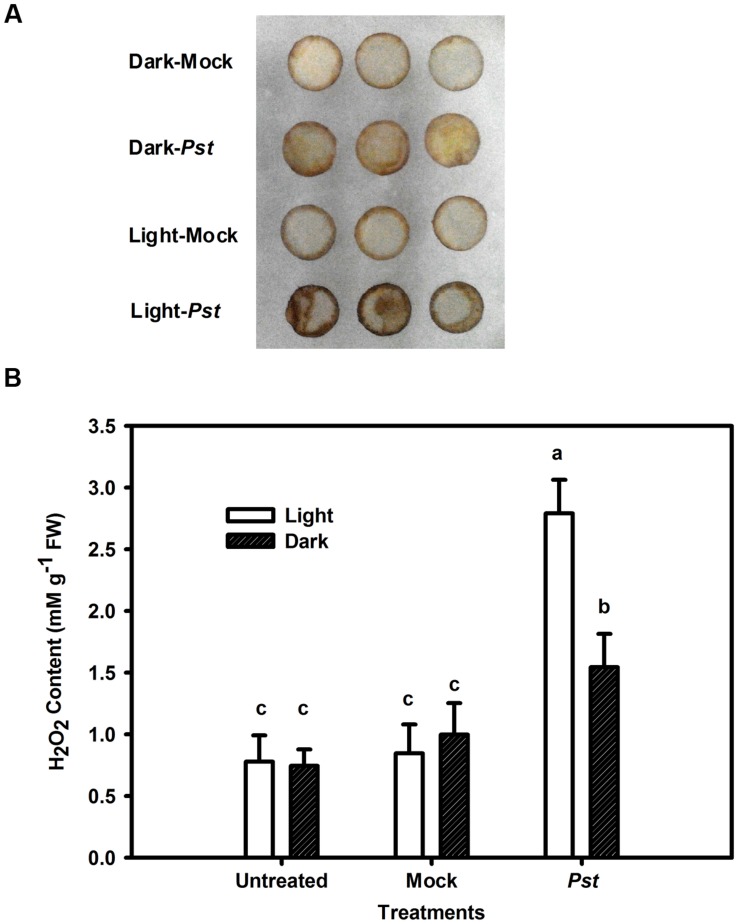
**Changes in H_2_O_2_ content in tobacco leaves after *Pst* infection.** **(A)** Histochemical detection of H_2_O_2_ accumulation using DAB staining in tobacco leaves after infiltration with distilled water (mock) or *Pst* under light (200 μmol m^-2^ s^-1^) or dark conditions. **(B)** The H_2_O_2_ content in tobacco leaves at 3 dpi of *Pst* under light or dark conditions. Means ± SE of six replicates are presented. Different letters above the columns indicate significant differences between treatments at *P* < 0.05.

**FIGURE 6:**
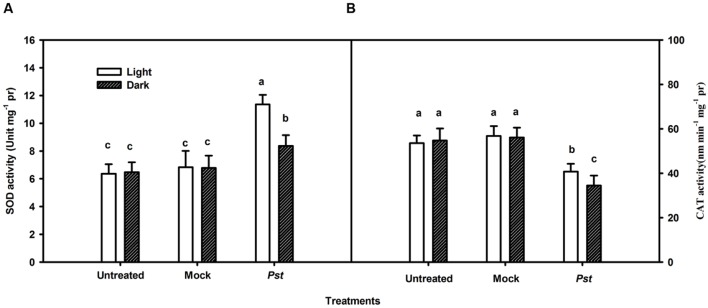
**Total cellular superoxide dismutase **(A)** and catalase **(B)** activities in tobacco leaves after *Pst* infection.** Means ± SE of three replicates are presented. Different letters above the columns indicate significant differences between treatments at *P* < 0.05.

Although light had no direct effect on *Pst* multiplication (**Figure 4**), light may influence ROS production in tobacco leaves after *Pst* infection. Because H_2_O_2_ is the most stable ROS that can be readily measured (Fleury et al., 2002), H_2_O_2_ production was evaluated in the *Pst*-infiltrated zone at 3 dpi. As shown in **Figure 5A**, *Pst* infection clearly enhanced H_2_O_2_ accumulation in tobacco leaves under light or dark conditions, with the content being increased by 258 and 107% at 3 dpi compared to untreated leaves under light and dark conditions, respectively (**Figure 5B**). The results indicated that a significant amount of H_2_O_2_ accumulated in tobacco leaves due to *Pst* infection under light and, to a lesser extent, dark conditions. As shown in **Figure 6A**, the activities of SOD increased in tobacco leaves at 3 dpi. In contrast, the CAT activity was greatly reduced after *Pst* infection (**Figure 6B**).

### The Effect of Exogenous H_2_O_2_ Pretreatment on the Bacterial Population in Tobacco Leaves after *Pst* Infection under Dark Conditions

**FIGURE 7:**
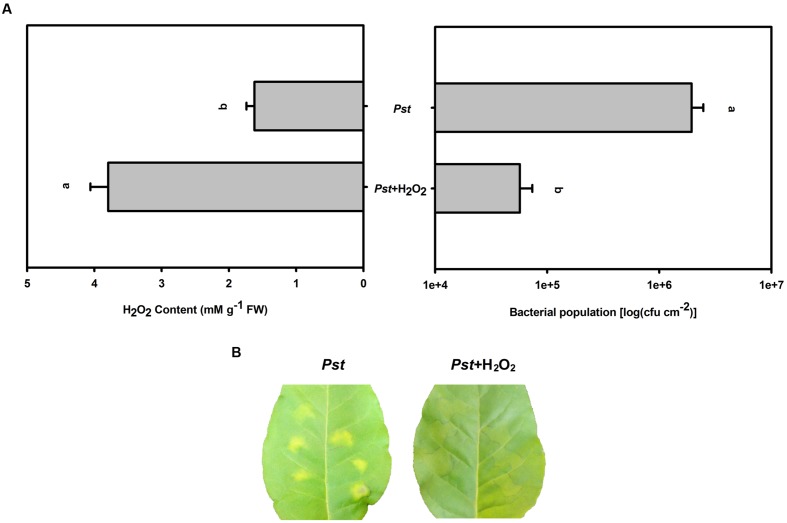
**The effect of H_2_O_2_ pretreatment on the bacterial population after *Pst* infection in tobacco leaves under dark conditions.** **(A)** H_2_O_2_ accumulation and the *Pst* population in tobacco leaves after pretreatment with exogenous H_2_O_2_ prior to *Pst* infiltration under dark conditions. Means ± SE of six replicates are presented. Different letters above the columns indicate significant differences between treatments at *P* < 0.05. **(B)** Representative images of changes in tobacco leaves pretreated with exogenous H_2_O_2_ at 3 dpi under dark conditions.

To successfully induce further H_2_O_2_ accumulation after *Pst* infection under dark conditions, exogenous H_2_O_2_ was uniformly sprayed onto tobacco leaves immediately prior to *Pst* infiltration. This H_2_O_2_ pretreatment alleviated chlorotic lesions and decreased the *Pst* population at 3 dpi under dark conditions (**Figure 7**).

### The Effect of MV or DCMU Pretreatment on the Bacterial Population in Tobacco Leaves after *Pst* Infection under Light Conditions

**FIGURE 8:**
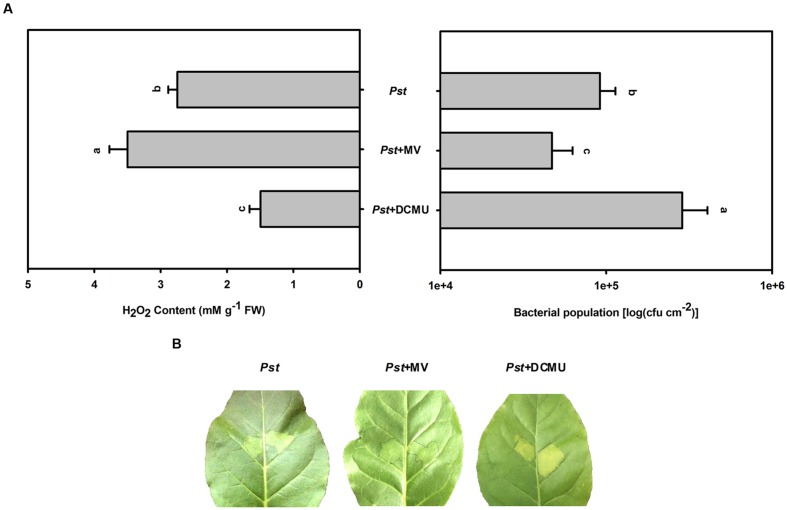
**The effect of MV or DCMU pretreatment on H_2_O_2_ accumulation and the *Pst* population after *Pst* infection in tobacco leaves under light conditions.** **(A)** The H_2_O_2_ accumulation and the *Pst* population in tobacco leaves after pretreatment with MV or DCMU prior to *Pst* infiltration under light conditions (200 μmol m^-2^ s^-1^). Means ± SE of six replicates are presented. Different letters above the columns indicate significant differences between treatments at *P* < 0.05. **(B)** Representative images of changes in tobacco leaves pretreated with MV or DCMU at 3 dpi under light conditions.

During illumination, MV preferentially accepts electrons from PSI and donates them to molecular oxygen, generating superoxide, which rapidly dismutates to H_2_O_2_ and ultimately results in H_2_O_2_ accumulation in chloroplasts (Halliwell, 1981). The herbicide DCMU is an inhibitor of electron transport from the primary quinone electron acceptor to the secondary quinone electron acceptor in PSII, which can decrease H_2_O_2_ production in chloroplasts (Marshall et al., 2002). To assess whether the size of the *Pst* population depends on the degree of H_2_O_2_ accumulation under light conditions, MV or DCMU pretreatment was used to increase or decrease H_2_O_2_ accumulation in tobacco leaves after *Pst* infection, respectively. MV pretreatment alleviated chlorotic lesions and decreased the *Pst* population at 3 dpi (**Figure 8**), whereas DCMU pretreatment aggravated chlorotic lesions and increased the *Pst* population (**Figure 8**).

## Discussion

In this study, we evaluated the role of light in *Pst* multiplication during the tobacco–*Pst* interaction in terms of its impact on ROS production. Less damage to the photosynthetic apparatus and reduced bacterial abundance were observed at 3 dpi under light conditions compared to darkness (**Figures 2** and **3**). Therefore, it is reasonable to speculate that the attenuation of damage to the photosynthetic apparatus resulted from the smaller bacterial population size observed at 3 dpi under light conditions. There are reports that light-mediated or light-facilitated signaling may play a key role in the defense response of plants to pathogens (Kangasjärvi et al., 2012). For example, Chandra-Shekara et al. (2006) revealed that resistance to turnip crinkle virus and tobacco mosaic virus in *Arabidopsis* and tobacco, respectively, is influenced by light. Pennypacker (2000) showed that reduced light led to increases in *Sclerotinia sclerotiorum* infection in soybean (*Glycine max*) and *Verticillium alboatrum*. Leone et al. (2014) reported that light plays an important role in regulating the trade-off between growth and defense that occurs in the shadow avoidance syndrome. The findings of host–pathogen interaction studies using *Arabidopsis* or rice (*Oryza sativa*) and bacteria also support the view that light-mediated or light-facilitated signaling is required for resistance (Guo et al., 1993; Genoud et al., 2002; Zeier et al., 2004). Moreover, light is required for SA biosynthesis and controls SA perception (Griebel and Zeier, 2008), and SA-associated defenses are effective at restricting invasion by biotrophic and hemibiotrophic pathogens (Glazebrook, 2005). Thus, light-controlled activation of the responses mentioned above is maybe one of the reasons of the reduced damage induced by *Pst* infection under light conditions.

As it is for plants, light is also a dominant environmental component of certain acclimatory and developmental processes in many pathogens. *P. syringae* pv. *tabaci* has three photosensory proteins and two bacteriophytochromes that serve as photoreceptors for visible light (Mandalari et al., 2013; Wu et al., 2013). Therefore, light may have a direct effect on *Pst* multiplication which ultimately led to the smaller bacterial population at 3 dpi in tobacco leaves under light conditions (**Figure 3**). However, no difference in the *Pst* population was observed between light and dark conditions after 3 days of cultivation in the absence of leaf tissue (**Figure 4**), indicating that the smaller bacterial population observed in leaves under light conditions was not caused by a direct effect of light on *Pst* growth.

Several reports have suggested that ROS accumulation is involved in plant defense responses (Murata et al., 2007). The direct reduction of oxygen to superoxide by reduced donors associated with PSI occurs during the Mehler reaction (Asada, 1999), and the photosystems of plant leaves are likely to be affected by ROS accumulation during stress (Takahashi and Murata, 2008; Mur et al., 2010). The observed reduced photosystem damage and the greater degree of H_2_O_2_ accumulation under light conditions compared to darkness (**Figures 2** and **5**) indicated that H_2_O_2_ accumulation was not the main reason for the photosynthetic apparatus damage induced by *Pst* infection in tobacco leaves. Besides, PSI attack by ROS occurs only if iron-sulfur centers can be maintained in a reduced state, which requires visible light (Sonoike et al., 1997). However, greater damage to PSI occurred in the dark, further supporting the viewpoint mentioned above. During plant–pathogen interactions, both the pathogen and the plant photosynthetic apparatus can be affected by ROS accumulation. Indeed, it has been reported that a greater abundance of H_2_O_2_ may lead to increased production of highly reactive hydroxyl free radicals via the Fenton reaction, with a toxic effect on the pathogen (de Torres Zabala et al., 2015). A smaller *Pst* population and a greater H_2_O_2_ accumulation were observed at 3 dpi in tobacco leaves under light conditions (**Figures 3** and **5**). Overall, The results indicated that greater H_2_O_2_ accumulation in the light after *Pst* infection in tobacco leaves led to a reduced impact of *Pst* on the photosynthetic apparatus, the mechanism of which may be associated with the increased generation of OH^⋅^ due to abundant H_2_O_2_.

To verify whether light suppresses the *Pst* population in tobacco leaves through H_2_O_2_ accumulation or a direct effect of the light itself, exogenous H_2_O_2_ was used to further increase the H_2_O_2_ content after *Pst* infection under dark conditions. The results showed that exogenous H_2_O_2_ pretreatment alleviated chlorotic lesions and decreased the *Pst* population at 3 dpi (**Figure 7**). In addition, MV or DCMU pretreatment was used to further increase or partially inhibit H_2_O_2_ accumulation in tobacco leaves after *Pst* infection under light conditions. We found that MV pretreatment alleviated chlorotic lesions and decreased the *Pst* population in the infiltrated zone at 3 dpi, whereas DCMU pretreatment aggravated chlorotic lesions and increased the *Pst* population (**Figure 8**). The results indicated that the chlorotic symptoms and the *Pst* population are negatively correlated with H_2_O_2_ accumulation; light itself had no effect, yet light did suppress the *Pst* population in tobacco leaves via H_2_O_2_ accumulation during infection.

In this study, light was observed to play an important role in the tobacco defense system by suppressing the *Pst* population through H_2_O_2_ accumulation. However, Zurbriggen et al. (2009) reported that transgenic tobacco plants are unable to generate high levels of ROS in chloroplasts and display attenuated cell death upon infection by the non-host pathogen *Xanthomonas campestris*. Thus, further studies are needed to thoroughly understand the mechanism of the effect of light on tobacco–*Pst* interaction with regard to H_2_O_2_.

## Author Contributions

D-DC, Z-SZ, G-YS, X-BS, and MZ designed the study. D-DC and M-JL carried out most of the experiments and data analysis. D-DC, Z-SZ, WC, and Y-BH wrote the paper. All authors read and approved the final manuscript.

## Conflict of Interest Statement

The authors declare that the research was conducted in the absence of any commercial or financial relationships that could be construed as a potential conflict of interest.

The reviewer JN and handling Editor declared their shared affiliation, and the handling Editor states that the process nevertheless met the standards of a fair and objective review

## Abbreviations

CAT: catalase
cfu: colony forming units
DAB: 3,3′-diaminobenzidine
DCMU: 3-(3,4-dichlorophenyl)-1,1-dimethylurea
dpi: days post inoculation
*F*_o_ and *F*_m_: initial and maximum fluorescence
*F*_v_*/F*_m_: maximal quantum yield of PSII
HR: hypersensitive reaction
H_2_O_2_: hydrogen peroxide
MR_820 nm_: modulated reflected signal of 820 nm
MV: methyl viologen
PRI: the photochemical reflectance index
PSI: photosystem I
PSII: photosystem II
*Pst*: *Pseudomonas syringae* pv. *tabaci*
*Pto*: *Pseudomonas syringae* pv. *tomato* DC3000
ROS: reactive oxygen species
SA: salicylic acid
SOD: superoxide dismutase
